# Chasing the Rainbow: The Non-conscious Nature of Being

**DOI:** 10.3389/fpsyg.2017.01924

**Published:** 2017-11-14

**Authors:** David A. Oakley, Peter W. Halligan

**Affiliations:** ^1^Division of Psychology and Language Sciences, University College London, London, United Kingdom; ^2^School of Psychology, Cardiff University, Cardiff, United Kingdom

**Keywords:** consciousness, suggestion, volition, agency, evolution, social

## Abstract

Despite the compelling subjective experience of executive self-control, we argue that “consciousness” contains no top-down control processes and that “consciousness” involves no executive, causal, or controlling relationship with any of the familiar psychological processes conventionally attributed to it. In our view, psychological processing and psychological products are not under the control of consciousness. In particular, we argue that all “contents of consciousness” are generated by and within non-conscious brain systems in the form of a continuous self-referential personal narrative that is not directed or influenced in any way by the “experience of consciousness.” This continuously updated personal narrative arises from selective “internal broadcasting” of outputs from non-conscious executive systems that have access to all forms of cognitive processing, sensory information, and motor control. The personal narrative provides information for storage in autobiographical memory and is underpinned by constructs of self and agency, also created in non-conscious systems. The experience of consciousness is a passive accompaniment to the non-conscious processes of internal broadcasting and the creation of the personal narrative. In this sense, personal awareness is analogous to the rainbow which accompanies physical processes in the atmosphere but exerts no influence over them. Though it is an end-product created by non-conscious executive systems, the personal narrative serves the powerful evolutionary function of enabling individuals to communicate (externally broadcast) the contents of internal broadcasting. This in turn allows recipients to generate potentially adaptive strategies, such as predicting the behavior of others and underlies the development of social and cultural structures, that promote species survival. Consequently, it is the capacity to communicate to others the contents of the personal narrative that confers an evolutionary advantage—not the experience of consciousness (personal awareness) itself.

## Overview

Most of us believe that what we call “consciousness” is responsible for creating and controlling our mental processes and behavior. The traditional folk usage of the term “consciousness” arguably has two aspects: the experience of “consciousness” and the contents of “consciousness”, our thoughts, beliefs, sensations, percepts, intentions, sense of agency, memories, and emotions. Over the past 30 years, there has been a slow but growing consensus among some students of the cognitive sciences that many of the contents of “consciousness,” are formed backstage by fast, efficient non-conscious systems.

In our account, we take this argument to its logical conclusion and propose that “consciousness” although temporally congruent involves no executive, causal, or controlling relationship with any of the familiar psychological processes conventionally attributed to it. In particular, we argue that all “contents of consciousness” are generated by and within non-conscious brain systems in the form of a continuous self-referential personal narrative that is not directed or influenced in any way by the “experience of consciousness” (which we will refer to as “personal awareness”). In other words, all psychological processing and psychological products are the products of fast efficient non-conscious systems.

The misconception that has maintained the traditional conscious-executive account largely derives from the compelling, consistent temporal relationship between a psychological product, such as a thought, and conscious experience, resulting in the misattribution that the latter is causally responsible for the former. Perceiving such relationships as causal in physical and social contexts is of course helpful and important, allowing humans to interpret events in our environment, particularly when describing and understanding predictive and goal-directed actions (e.g., Blakemore et al., 2001; Woods et al., 2014). When we witness two billiard balls collide, we intuitively perceive one ball forcing the other to move in a designated direction despite simply observing a sequence of events. As Hood (2006) points out “*humans are causal determinists; we cannot help but experience the world as a continuous sequence of events and outcomes*.” Spatial continuity and temporal contiguity increase the likelihood that we will perceive causality (e.g., Woods et al., 2014). However, while two events can be temporally and spatially contiguous, we argue that personal awareness is qualitatively distinct and separate and as such does not exert any causal influence over the contents of the personal narrative (Halligan and Oakley, 2000; Blackmore, 2012, 2016). In other words, despite its intuitive attractiveness and folk acceptance, the ascription of executive functions or agency to “consciousness” either in part or as a whole, or to the “experience of consciousness,” we claim is a misconception.

Consequently, the focus of this paper is less concerned with explaining personal awareness, which we take as a given, but more with explaining the properties, functions, and adaptive significance of the non-consciously generated, self-referential psychological content of the personal narrative. This conceptual decoupling, we suggest, offers a more productive starting point and focus for cognitive science when exploring the origin and function of psychological processes, and the control over them which was previously attributed in large or small part to the presence of an executive “consciousness.” Moreover, we consider that it is the capacity to share the contents of the non-consciously generated personal narrative stream, rather than personal awareness *per se*, that confers an evolutionary advantage. The potential to share selective psychological content from the personal narrative, such as ideas and knowledge, underpins the development of socially adaptive strategies including understanding and predicting the behavior of others, and ultimately cultural evolution.

Notwithstanding the above, we have little option but to use in this article the terms “consciousness,” “experience of consciousness,” “conscious awareness,” and “contents of consciousness” (all with single quotation marks) when referring to the traditional hybrid construct that implies some functional dependency between personal awareness and the control of higher psychological processes. Ultimately by removing what we see as the mistaken attribution of executive control and agency to “conscious experience,” we hope to avoid the necessity of characterizing cognitive/psychological processes in terms of the traditional binary distinction of “conscious” vs. “unconscious.” With this in mind, we favor the use of “psychological,” as the more neutral term in relation to this distinction, in preference to “cognitive.” Similarly, we use the term “non-conscious” in preference to “unconscious,” to reflect our view that all psychological processing and processes, including those forming what we call the personal narrative, occur outside “conscious experience.” Seen in such a light, a major aspect of the “hard problem of consciousness” (the problem of trying to explain how phenomenal experiences can influence physical processes in the brain) can be avoided in that the “experience of consciousness” (personal awareness) we argue can be seen to be a real, but passive emergent property of psychological processing and not some executive process capable of animating and directing our mental states. In this respect, we favor Huxley's analogy which regarded “consciousness” as being like a steam whistle on a train—accompanying the work of the engine but having no intrinsic influence or control over it (Huxley, 1874). In summary, personal awareness is real, present, and contemporaneous with non-conscious products, but it is not causal and does not exert any influence on our psychological products. Our account does not aim to explain, the other feature of the “hard problem”—namely the question as to why we have subjective experience at all.

In addition to presenting our view of “consciousness” in more detail in this paper we will discuss some of its broader implications for cognitive neuroscience. We will also explore its relevance in relation to the social role of suggestion, its potential for understanding of processes underlying suggestion, dissociation, and related clinical conditions, as well as implications for the topics of free-will and personal responsibility. We start however, with a brief historical overview of ideas about “consciousness.”

## The rise and fall of “consciousness”

In 1976, Jaynes suggested that early in human evolutional history, the experience of “consciousness” was initially interpreted as external voices that commanded actions and framed perceptions and beliefs not that dissimilar from hallucinations and delusions experienced in schizophrenia. More recent folk accounts of psychological states however have accepted “consciousness” as arising from, and under the control of, the individual's “self” (Bargh and Morsella, 2008). However, as far back as the nineteenth century, the founding fathers of psychology observed that many of our mental experiences arise from processes that we are not consciously aware of (James, 1892; von Helmholtz, 1897; Wundt, 1902). The latter realization, derived in part from observation of phenomena observed in hypnosis (Bargh and Morsella, 2008), was incorporated into the writings of Charcot and Freud (Oakley, 2012). This was further reinforced by the observations of several influential psychologists at the beginning of the “cognitive revolution” (Miller, 1962) who noted that that even a cursory introspective examination of one's own “conscious awareness” quickly revealed that the products of thinking and perception were the result of non-conscious processes (Nisbett and Wilson, 1977; Halligan and Oakley, 2000).

Nevertheless over the past 60 years, cognitive psychology has retained a distinction between “automatic” mental processes—not involving “conscious awareness” and “controlled” processes that did (Miller, 1962; Nisbett and Wilson, 1977; Kihlstrom, 1987; Gazzaniga, 1988; Moscovitch and Umiltà, 1991; Halligan and Marshall, 1997; Velmans, 2000; Driver and Vuilleumier, 2001; Wegner, 2002; Pockett, 2004; Hassin et al., 2005; Frith, 2007, 2010; Earl, 2014; Frigato, 2014). The Global Workspace theory (Baars, 1988, 1997) likened “consciousness” to a working theater where psychological events created by non-conscious processes taking place behind the scenes, allowed some to enter onto the stage of “conscious awareness.”

This long standing and intuitive account of consciously mediated executive control has however been challenged, by a small but growing number of students of neuroscience (Gazzaniga, 1988, 2000; Haggard and Eimer, 1999; Halligan and Oakley, 2000; Velmans, 2000; Wegner, 2002; Gray, 2004; Pockett, 2004; Frith, 2007, 2010; Baumeister and Bargh, 2014; Frigato, 2014) who demonstrated the involvement of ever more sophisticated non-conscious systems involved in the execution and co-ordination of complex and interdependent psychological functions underlying thought, motivation, decision making, mathematical ability, and mental control in the pursuit of goals (Dijksterhuis and Aarts, 2010; Hassin, 2013).

Recognition of the pervasive adaptiveness of non-conscious systems increased further over the past 10 years (Bargh and Morsella, 2008) with non-conscious mechanisms being increasingly implicated in more complex phenomena, such as decision-making, face perception, conformity, and behavioral contagion (Hassin et al., 2005; Bargh et al., 2012), to the point where it was claimed that non-conscious systems could carry out all of the psychological activities traditionally assumed to depend on “consciousness” (Hassin, 2013). Consistent with the latter view, it has been argued that conscious control of behavior was purely illusory (Wegner, 2002). Not all researchers and theorists however agree and some form of executive role for “consciousness” systems continues to be retained or emphasized (Baumeister et al., 2011; Frith and Metzinger, 2016).

In parallel with these developments in cognitive psychology, compelling complementary evidence from cognitive neuropsychology has begun to highlight some of the fault lines between traditional accounts of “conscious” and “unconscious processes.” For example, patients with “blindsight” following damage to primary visual cortex show that actions can be guided by sensory information that they remain largely unaware of, challenging the common belief that perceptions must enter “conscious awareness” to affect or produce our actions (Weiskrantz, 1985). Similarly in cases of visual neglect where, patients can show impressive non-conscious processing for stimuli on the neglected side of their visual fields, including object identification despite lack of reported visual awareness (Marshall and Halligan, 1988; Driver and Mattingley, 1998).

## Quantifying the timing of “conscious awareness”

In the 1980's powerful evidence emerged where it was shown that our intentions to act (deliberately make a motor movement) occurred later than the ongoing preparatory brain activity (readiness potentials) in motor systems of the brain (Libet et al., 1983). This implied that awareness of the decision to move and preparation of that movement was produced by prior non-conscious processes with the experience of conscious intention coming too late to be the initiator of the motor act. Further evidence that timing of the readiness potential and experience of the intention to move was non-linear, suggested that the two were largely independent (Haggard and Eimer, 1999; Schlegel et al., 2013). Also, research using hypnotic suggestion to create self-initiated movements without the conscious experience of intention showed that unintended, “involuntary” movements were also preceded by readiness potentials (Schlegel et al., 2015) but that the estimated time of the movements obtained from the participant was more consistent with passive rather than with voluntary movements (Haggard et al., 2004; Lush et al., 2017).

Given the independence of readiness potentials and the experience of an intention to act, one possible conclusion is that the latter is not part of the stream of processing leading to a movement, but rather the result of a consistent (non conscious) *post-hoc* attribution of intentionality to any non-reflexive, self-generated action. EEG evidence investigating phantom limb movement also indicated that the experience of both positive and negative volition is generated by brain activity occurring *before* the movement itself (Walsh et al., 2015a).

Clearly there are processes involved in what are described by the individual as voluntary movements that are upstream of the readiness potentials, but there is no reason to assume that any of these processes are not also non-consciously produced. Overall, the evidence appears consistent with the view that preparation to move originates in non-conscious systems and that the awareness of the intention to move is experienced only if that preparation becomes part of an ongoing, non-consciously generated personal narrative.

Consistent with this is a review of evidence from studies of brain damage leading to spatial neglect, which has distinguished widespread areas of the brain capable of processing up to eight different aspects of spatial perception (such as image perception, spatial image positioning, and emotions related to the images) and two areas (anterior cingulate and precuneus-posterior cingulate) involved in access to “consciousness” (Frigato, 2014). This suggests that brain injury can damage aspects of perception or can interfere with “consciousness” associated access mechanisms, preventing the consciously correlated experience of certain types of percept whilst leaving access to these perceptual processes at a non-conscious level intact. Importantly, however, brain processes taking place in both the access areas and the perceptual areas can be regarded as non-conscious, with the “access areas” responsible for selectively forming the products of the perceptual processing areas into a personal narrative. It is only the personal narrative, we argue, that is accompanied by personal awareness.

Despite increasing, persuasive evidence from psychological and neuropsychological research over the past 30 years demonstrating the involvement of non-conscious processes in generating the “contents of consciousness,” there has been a widespread reluctance to draw the natural conclusion that both aspects of “consciousness” (experience and contents) depend on non-conscious mental processes. The intuitive preference for retaining a conscious-experience led model of mental processing is supported by long-standing beliefs, nurtured by daily experiences whereby “self” and “consciousness” are inextricably linked to all forms of perception and motor control.

However, we argue that attributing psychological/executive functions to “conscious experience” (personal awareness) contributes little to the explanatory account of the processes responsible for our ongoing stream of psychological states.

In particular, we include all contents of “consciousness” such as intentions, the perception of self, and the experience of executive control, as products of non-conscious processes. Non-conscious brain systems carry out all core biological processes and our account is consistent in suggesting that psychological functions, including those normally attributed to “consciousness” should be regarded as no different (Hassin, 2013). Non-conscious causation provides a more plausible (albeit non-intuitive) basis for explaining both what is conventionally considered to be “contents of consciousness” and the concurrent “experience of consciousness.” It is also consistent with the observation that, “*in the rest of the natural sciences, especially neurobiology, the assumption of conscious primacy is not nearly as prevalent as in psychology. Complex and intelligent design in living things is not assumed to be driven by conscious processes on the part of the plant or animal, but instead by blindly adaptive processes that accrued through natural selection”* (Bargh and Morsella, 2008, p. 8).

Also, in relation to social and cultural contexts, there is increasing evidence that non-conscious neural systems arrive pre-configured with developmentally receptive psychological tools designed to navigate social environments and challenges (Cosmides and Tooby, 2013). The ability to share the contents of our individual psychological states with others however confers a social benefit and a powerful evolutionary advantage (Jaynes, 1976; Humphrey, 1983; Barlow, 1987; Dunbar, 1998; Charlton, 2000; Velmans, 2000; Frith, 2007, 2010; Baumeister and Masicampo, 2010). In particular, we argue that it is precisely the capacity to communicate selectively the contents of our non-consciously generated personal narrative that confers an evolutionary advantage, and not the “experience of consciousness” *per se*.

## Anthropomorphism and the search for meaning

Having hopefully displaced “consciousness” from it's traditional executive driving seat, our account naturally begs the question as to its purpose or function, in particular, why did consciousness arise in evolving organisms if it doesn't appear to do anything? To address this, a consideration of the functional explanations offered for other apparently evident but equally mysterious phenomena may be helpful.

Rainbows result from the bending of sunlight passing through raindrops, which act like prisms to create a distinctive arc of colors in the sky, with red on the outer part and violet on the inner section. Despite appearances, the rainbow does not occupy a particular place, its apparent position depends on the observer's location in relation of the sun. Nevertheless, like “conscious experience,” rainbows are subjectively “real” phenomena produced by physical processes. However, before the physical explanation was discovered, many different cultures felt compelled to attribute a range of different functions or purposes to the existence of the rainbow phenomenon. For example, a biblical version regards rainbows as a sign from God to never again flood the earth and kill every living thing (Genesis 9:8–15). In Graeco-Roman mythology, the rainbow was considered to be a path between Earth and Heaven. In Chinese culture it was believed to be a slit in the sky sealed by a goddess using stones of five different colors. In Irish mythology, the point where the rainbow makes contact with the earth was said to indicate the elusive hiding place of a pot of treasure.

Most of these accounts can be seen as instances of a wider predisposition toward anthropomorphism, a predisposition to attribute intentions, beliefs, and characteristics to non-human and inanimate objects and events, which we would argue is deeply embedded in non-conscious psychological processes. Anthropomorphism itself can be seen as an example of a wider human “drive for causal understanding” (Gopnik, 2000) that can lead to confabulations and delusions in some neuropsychological conditions, and also in neurologically intact individuals (Coltheart, 2016), particularly given the apparent predisposition in humans toward abductive inference (Fodor, 2000). Gopnik (2000) suggests that “*explanation may be understood as the distinctive phenomenological mark of the operation of a special representation system*”. “*designed by evolution to construct …. “causal maps”…abstract coherent, defensible representations of the causal structure of the world around us … “as” the phenomenological mark of the fulfillment of an evolutionarily determined drive*”. The result is occasionally manifest in “magical, mythical, and religious explanations,” especially in situations where the alternative is having no explanation at all, but overall it is “*consistent with the view that the [representational] system evolved because, in general, over the long run, and especially in childhood, it gives us veridical information about the causal structure of the world*” (Gopnik, 2000, p. 315).

Rainbows and other celestial phenomena such as eclipses and the northern lights are indisputably as “real” as personal awareness. However, little is gained, by asking “*what is the purpose or function of an eclipse or a rainbow?*” Indeed, posing such a question assumes some hidden, significant explanation to be discovered. Importantly, in our view personal awareness, like rainbows and eclipses, is not a product of evolutionary selection processes and does not have a demonstrable evolutionary purpose in its own right. Rather it is the incidental accompaniment to the final stages of the information processes in the brain responsible for creating a personal narrative. In the same way that there is arguably no purpose to an eclipse or a rainbow, we suggest the same for personal awareness. Personal awareness just “is,” though as humans we feel compelled to “explain” it by attributing a functional capacity, purpose, or meaning to it and in so doing, we argue, has generated a host of misconceptions. In the case of “consciousness,” the exquisite temporal contiguity between personal awareness and the contents of the personal narrative have understandably and readily provided a reliable, intuitive and commonly unquestioned explanation for a compelling causal association between the two that remains particularly difficult to argue against.

The dangers of drawing such anthropomorphic attributions or explanations was nicely captured by Albert Einstein (quoted in Home and Robinson, 1995, p. 172): “*If the moon, in the act of completing its eternal way around the earth, were gifted with self-consciousness, it would feel thoroughly convinced that it was traveling its way of its own accord on the strength of a resolution taken once and for all*”. We should be wary of making the same mistake with consciousness.

A similar misattribution surrounds the experience of a phantom limb following amputation, often associated with pain and still considered by many as counter-intuitive and anomalous (Halligan, 2002). Historically, in keeping with religious beliefs at the time, this common phenomenological experience was initially explained as being the product of a miraculous form of limb restoration (Halligan, 2002). This explanation also avoided the necessity to challenge the compelling folk account that it was not possible to feel a body part that was no longer physically present. The source of this misconception was nicely addressed by Melzack (Melzack, 1992; Saadah and Melzack, 1994) who points out “*Phantoms become comprehensible once we recognize that the brain generates the experience of the body. Sensory inputs merely modulate that experience; they do not directly cause it*. p. 126” (Melzack, 1992).

## The oakley-halligan account

A key feature of our account (some of which has been anticipated by others) is that it does not set out to offer an explanation for the subjective “experience of consciousness” but rather to highlight what we consider to be the fundamental misconception rooted in everyday experience and embedded in the powerful folk-view of the nature of “consciousness.” Central to our view, developed over many years (Oakley, 1985, 1999a,b, 2001; Oakley and Eames, 1985; Halligan and Oakley, 2000; Brown and Oakley, 2004), is the simple proposition that all neuropsychological processing takes place independently of the experience of “consciousness.” This is not to deny the powerful and ubiquitous existence of “conscious experience” but rather to claim that all executive psychological processes irrespective of how quickly and intuitively causally they might appear, actually reflect background neuropsychological activity that takes place in non-conscious systems. As noted earlier, to avoid unwanted associations embedded in traditional accounts of “consciousness” we have choosen to use the terms “personal narrative” and personal “awareness” in our account in place of “contents of consciousness” and “experience of consciousness.”

**Figure 1:**
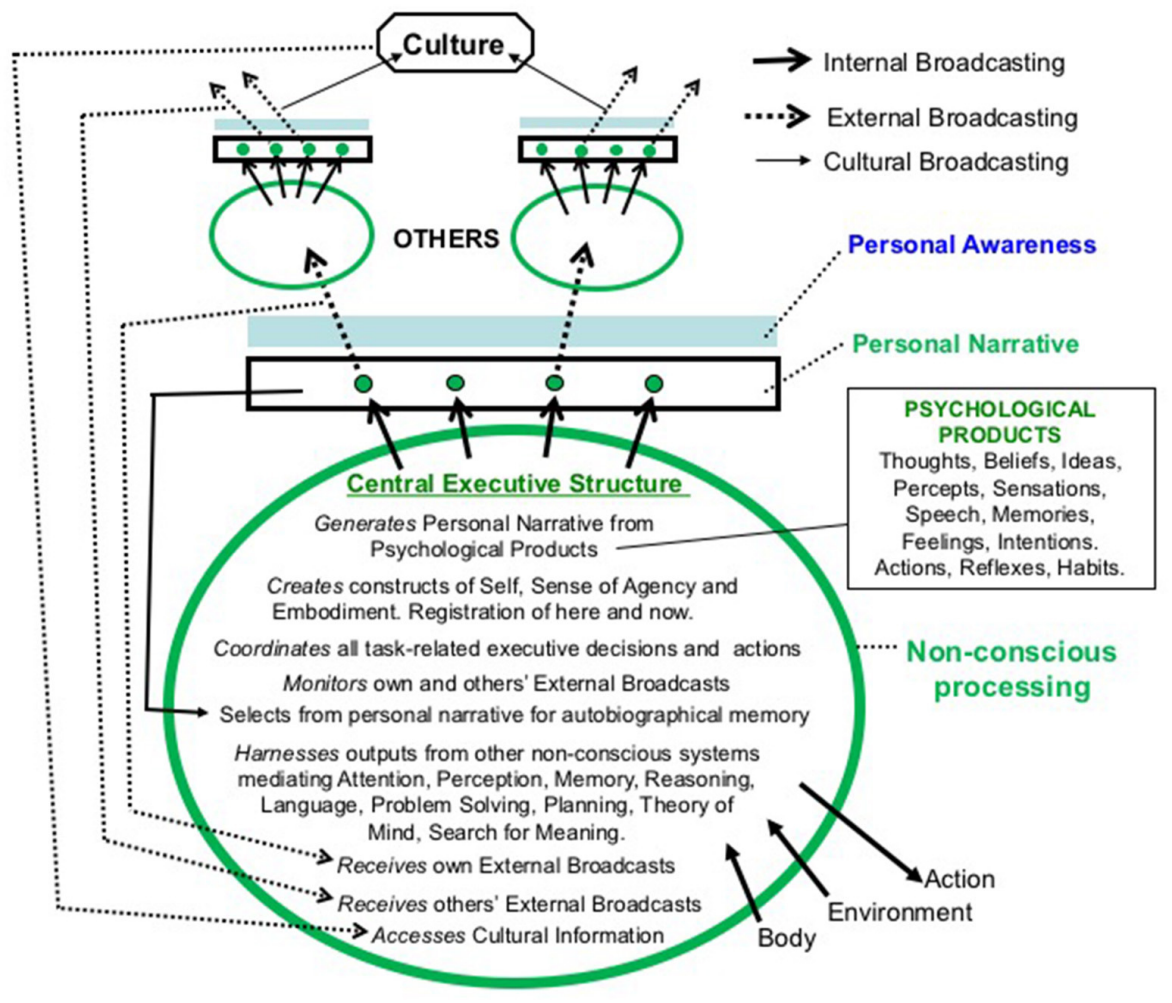
The Oakley-Halligan model. The schematic diagram shows all current CES functions and other psychological activities as non-conscious processes and their products. The most task-relevant of these psychological products are selected by a Central Executive Structure (CES) to create an ongoing personal narrative via the process of Internal Broadcasting. This personal narrative is passively accompanied by personal awareness - a by-product of Internal Broadcasting. Some components of this narrative are selected by the CES for further transmission (External Broadcasting) via spoken or written language, music, and art to other individuals. The recipients in turn transmit (internally then externally) their own narrative information, which may contain, or be influenced by, the narrative information they have received. The CES also selects some contents of the current personal narrative for storage in autobiographical memory. The contents of external broadcasts contribute (via Cultural Broadcasting) to an autonomous pool of images, ideas, facts, customs, and beliefs contained in folklore, books, artworks, and electronic storage systems (identified as “Culture” in the Figure) that is accessible to others in the extended social group but is not necessarily dependent on direct interpersonal contact. The availability of culturally based resources is a major adaptive advantage to the social group and ultimately to the species as a whole. The CES has access to self- and other-generated externally broadcast content as well as to cultural information and resources, all of which have the potential to provide information that supports the adaptedness of the individual and to be reflected in the contents of their personal narrative. As a passive phenomenon, personal awareness exerts no influence over the CES, the contents of the personal narrative or on the processes of External and Cultural Broadcasting. In the Figure non-conscious process are identified in green and personal awareness (subjective experience) in blue.

In our view (summarized in Figure 1), it is more parsimonious to conclude that personal awareness is a phenomenal accompaniment of a continuously updated, and individually-oriented *Personal Narrative*, produced and coordinated by extensive non-conscious systems forming a Central Executive Structure (CES) (Halligan and Oakley, 2000). This personal narrative represents a small, and selective fraction of the total products of psychological activity taking place in the brain and available to the CES.

The Personal narrative (PN) has compelling real-world face validity—particularly when linked to the notions of “self” and “personhood.” While previously attributed to “consciousness”—given its temporal association with the same—in our account PN is not produced or in any way constrained by conscious experience. Contents of the PN are however experienced by us as embodied individuals. All psychological products of mind are housed within a corporeal framework which ensures that the PN provides meaning for what is happening and preparedness for all embodied action options (movements, gestures, verbalizations, actions) that from a subjective perpective are purely private and publicly inaccessible. This sense of embodiment and meaning is critical to the non-conscious nature of the contents of the PN and forms the gravitational focus of a “psychological self” located within a “bodily self.”

We describe the process of generating this personal narrative as *Internal Broadcasting*. This process, which we previously referred to as “outing” (Halligan and Oakley, 2000) is similar to other accounts which suggest that there is a special brain function, a form of “interpreter” (Gazzaniga, 1985), which constructs a meaningful account of our non-consciously generated behavior and provides an ongoing explanation for it through a process of “narratization” (Jaynes, 1976). In our account the self-referential personal narrative, created as a product of internal broadcasting from non-conscious systems, is accompanied by personal awareness. In Figure 1, non-conscious processes relating to CES activity including those responsible for the reiterative creation of the personal narrative are shown in the oval “bubble.” The non-conscious end-products of CES activity that form the personal narrative are shown in the rectangle immediately above the bubble. It is important to highlight that the personal narrative (as an output) has no processing capacity of its own, it is simply the end-product of selective, competitive psychological processes. “Personal awareness” is represented by the separate, filled rectangle immediately above the personal narrative. As we discuss elsewhere, we argue against any functional or causal relationship between the personal narrative and personal awareness.

A central role of the CES involves the selection from a wide range of available psychological products those that best reflect ongoing brain actvity in relation to current tasks, facilitating identification of the most relevant behaviors for an individual to engage in, and the choice of the most appropriate actions. The CES draws from these competing sources to create a personal narrative relevant to current needs, although other high-priority brain events may also be represented in the narrative in the form of non-task related thoughts, memories, and emotions such as intrusive reflexive responses, emotional responses, traumatic memories, and actions not as planned, originating outside the CES. Importantly, however, most brain activity, including much of that taking place in the CES, is not represented in the personal narrative. Typically, not included are processes that underlie most basic bodily functions regulated by the CNS, such as breathing, the control of individual muscles, digestion, the onset of sleeping, and waking up [or of events that take place between the two, such as dreaming or the processing, reorganizing, and consolidation of vocabulary and memories (Rasch and Born, 2013; James et al., 2017)]. Also there is no record of the brain activity that underlies the identification of sounds, sights, tastes, smells, and the integration of these into the changing sequence of events and objects in the outside world, or of processes underlying thoughts, actions, likes and dislikes, feelings, and moods. The CNS selects relevant end products from these psychological processes when creating the personal narrative, but typically includes no reference to the how these products were generated. In many situations involving rapid or routine decision making for example, underlying thought processes are not reflected in the personal narrative. Hovever, if the process of making a decision or thinking about a problem becomes part of the task in hand, many of these underlying thoughts may be internally broadcast to form part of the ongoing narrative and hence are accompanied by personal awareness (a parallel “conscious” experience), a distinction that Kahneman (2011) drew between “fast” and “slow” thinking.

Importantly, our account is consistent with phenomenological reality. For instance, I don't know what I am going to say or write next—it simply appears as a thought or verbalization. The personal narrative is not the originator but rather the vehicle through which such non-conscious products are presented. This point was dramatically illustrated by the children's author Enid Blyton, who described how, when beginning a new book she would simply sit at her typewriter and wait, and then “My hands go down on my typewriter keys and I begin. The first sentence comes straight into my mind, I don't have to think of it.….To write book after book without knowing what is going to be said or done sounds silly—and yet it happens. Sometimes a character makes a joke, a really funny one, that makes me laugh as I type it on my paper—and I think, ‘Well, I couldn't have thought of that myself in a 100 years!’ and then I think, ‘Well who *did* think of it then?’” (Stoney, 1992, p. 216–217). Equally, there is anecdotal and research evidence that apparently spontaneous acts of creativity in science and art arise through non-conscious processes, as with recalling the maiden name of our mother, the results of which are later incorporated fully-formed into the personal narrative, often after a period of sleep or distraction (Ghiselin, 1952; Miller, 1962; Ritter et al., 2012). This is not to deny that they may then be further refined or incorporated, by equally non-consciously generated thought processes, if they become part of the ongoing task represented in the personal narrative.

An integral and key aspect of the personal narrative process, we argue, is the incorporation of a self-referential perspective. This provides for the sense of agency and autobiographical time, as well as ownership and responsibility for what are considered to be our internally generated thoughts, actions, percepts, sensations etc. Agency in the context of movement is preserved in the personal narrative by the introduction of the representation of an intention to act in close temporal proximity to the relevant body part movement. This coherence is important for maintaining a consistent, meaningful personal narrative where the notion of self is represented as being the key reference for executive control. It is also consistent with the observation that neural indicators of an impending movement precede the appearance of the intention to move in the personal narrative.

The CES monitors and, where necessary, amends the contents of the personal narrative on an ongoing basis to ensure current and retrospective consistency in relation to self over time and to avoid and resolve internal conflicts (cognitive dissonance). Importantly the ongoing personal narrative (comprising thoughts, beliefs, ideas, intentions, perceptions, feelings etc.,) is available for storage in whole or in part in episodic/autobiographical memory systems and these serve in turn as an important reference point for future action. In this sense, episodic memory is based on a current account of events (the personal narrative) created by the CES, colored and shaped by individual needs, beliefs and goals and forms the basis on which the past is represented and on which current beliefs, behavior and thoughts can be justified, particularly in interaction with other individuals.

Finally, we propose that the creation of this consistent personal narrative confers an evolutionary advantage for the individual in the form of survival and reproductive benefits through the ability to selectively share its contents, and via a potentially wider benefit for the human species as a whole (Wilson, 1975; Dawkins, 1976; Halligan and Oakley, 2015). The social advantage would be expected occur initially within families and near relatives, extending to progressively wider groups with close genetic relationships. We refer to the first stage of the process of sharing narrative information with other individuals as *External Broadcasting*. This involves the transmission of private mental psychological contents, such as thoughts, ideas, concepts, beliefs, abstractions, sensations, feelings, urges, and concerns from the personal narrative, implicitly via facial expression, posture and gestures but, most importantly, conventionally through speech and other means, such as writing, art, music and electronic media. The CES also has access to shared information deriving through channels emphasized in social mirror theory, such as song, dance, and various forms of play, especially that involving make-believe and role-taking (Whitehead, 2001).

A further, third stage, *Cultural Broadcasting*, is the process by which information, thoughts and ideas enter a communal or social pool (labeled “Culture” in the figure) which is not dependent on direct contact between individuals and is represented in written or digital materials, artifacts, and social structures.

Individuals receive both their own and others external broadcasts via relatively autonomous (modular) lower-level perceptual and sensory systems. An important role of the CES involves monitoring both of these inputs to incorporate relevant information from external broadcasts of others into its own ongoing processing and in the case of the individual's own external broadcasts to correct or update earlier transmitted information if necessary. Individual reasoning is largely intuitive, self-centric, and biased in favor of existing beliefs (Mercier and Sperber, 2017), but in social contexts, while individuals seek to confirm their own viewpoint through argumentation, they can be exposed to conflicting views of others via the process of mutual external broadcasting and can critically assess them, leading ultimately to the development and circulation of better-formulated social policies and scientific beliefs.

The CES is also able to access cultural information. In terms of the model we are presenting, “Culture” has a dynamic element in that it originates in, and is mediated initially by, individual External Broadcasts. More importantly, it comprises a supra-individual system (artifacts, books, internet etc.,) accessible directly by individuals via downstream non-conscious systems and thereby upwardly available to the individual's CES and may ultimately be reflected in the content of their personal narrative. Cultural Broadcasting is a one-way process. “Culture” is being fed into via the external broadcasts of individual personal narratives but it attains an independent status a resource or a context that is accessible to individuals rather than being actively outputted to them.

While both External and Cultural Broadcasting are supra-individual, it is important to emphasize that humans are highly adapted and indeed prone to take advantage of feedback they receive via external and cultural broadcasting from others and from their environments. Humans are equipped, for example, with inbuilt predispositions including the generation of a sense of agency, the tendency to infer causality from environmental and social events, to attribute human characteristics to non-human and inanimate objects and phenomena, to develop a Theory of Mind and to respond to interindividual influences such as instruction, suggestion, and the transmission of beliefs. We have considered some of these above and explore examples of adaptive receptivity further below. For now, however, it is important to note that in our view all of such adaptations are mediated solely by non-conscious processes.

## Similarities to other accounts

Currently influential psychological views of consciousness are broadly classifiable as global workspace and higher order theories. Representing the first of these is Baars (1997) “Theater of Consciousness.” Central to this metaphorical account is the view that within the brain there are neural areas that “work together to display conscious events” (p. ix) and to produce a coherent story—by analogy to the writers, directors, producers etc who are responsible for what occurs on stage. This has clear similarities to our “personal narrative” but in the “theater” account “consciousness” appears to be a distinct entity with a specific role, it “creates access to many knowledge sources in the brain” (p. 6). In our account the personal narrative (“contents of consciousness”) or personal awareness (“experience of consciousness”) are both end products of non-conscious processes and have no active role.

Higher order theories (see Carruthers, 2007) view consciousness as a property of a second more executive level of processing by which, for example, we not only perceive (say, the rainbow) but become aware of our perceptions (i.e., are aware of being aware of seeing a rainbow). In our model this second level of processing is represented in the non-consciously generated personal narrative independently of the parallel experience of consciousness. In common with our own account neither theater nor higher-order theories offer a solution to the “hard problem” of how the processes they propose produce a subjective/conscious experience (personal awareness) within a physical entity such as the brain.

As functionalists, the proponents of theater and higher-order theories could however argue that there is no need to distinguish a separate mental property (“personal awareness”) above and beyond the generic functional property that mental states are internal states of thinking creatures. As such, there is no hard problem to be solved. In our account, while never denying the phenomenal existence of consciousness (personal awareness), we adopt an epiphenomenalist view, whilst recognizing its acknowledged lack of intuitive appeal. We argue that subjective mental experiences are non-efficacious or “collateral” products of neurophysiological activity without an obvious proximal purpose in the same way that rainbows and eclipses are in relation to underlying physical processes. Nevertheless, we recognize that in the search for meaning, personal awareness as with eclipses and rainbows has been endowed variously by tradition and folklore with both a function and a capacity to interact.

In sum, we propose that consciousness (personal awareness) is a product of antecedent brain processes and has no functional role in itself for influencing subsequent brain states. As such, lacking an executive function, we consider the experience of consciousness as epiphenomenal. We accept that when we refer to, and talk about, personal awareness this reference is not caused by personal awareness itself but is part of the narrative generated directly by ongoing neural processes. For our part we defer the hard problem on the assumption that ultimately cognitive neuroscience, information theory and related disciplines will identify the processes that are accompanied by subjective experience and provide some insight into the underlying mechanisms creating the rainbow that is conscious experience.

There are also some similarities between the model we present, and other recently published theoretical views. For example the Passive Frame Theory (Morsella et al., 2016a,b), argues that the contents of “consciousness,” including a self-focused narrative, are generated by non-conscious processes with awareness of these contents being a later arriving accompaniment. This account however goes on to conclude, however, that “consciousness” serves an intrapersonal role, critical for the functioning of the skeletal muscle output system. By contrast, in our model we propose that the main advantage of creating a self-referential personal narrative is a social one deriving from the ability to share its contents with others. Pierson and Trout (2017) also emphasize the intra-personal function of consciousness, describing the experience of consciousness in particular as an evolved force separate from brain function that underlies volition and free-will especially in relation to movement. In their view, consciousness can exert an active downward influence on brain processes, in particular it can initiate volitional movements, which are then executed by non-conscious processes in the brain. Though the authors present a case for why “consciousness” evolved they accept that there is at present no explanation of the mechanism by which an apparently non-physical “consciousness” could be created in living systems. The latter is consistent with our own epiphenomenalist stance, and the view that the experience of consciousness is devoid of any executive capabilities. In contrast, a proposed data compression approach to understanding the phenomenon of “consciousness” derived from theoretical computer science (Maguire et al., 2016), emphasizes the social relevance and evolutionary advantage deriving from the development of adaptive strategies including the ability to predict the behavior of others based on a strong representation of the self. A similar information processing account (“attention schema theory”) proposed by Graziano and Webb (2017), views the experience of consciousness as linked to the development over a long evolutionary period of a self-referential internal model of awareness. In common, with our account the attention schema model presents conscious experience as an accompaniment but in contrast does not address the contents of consciousness. Also, the relationship we propose between the personal narrative and episodic memory has a number of points in common with the views of Mahr and Csibra (2017), particularly in relevance to social interaction.

## The construct of “self”

According to Damasio (2003) the self “*is not a thing but a process, one that produces phenomena ranging from the very simple (the automatic sense that I exist separately from other entities) to the very complex (my identity, complete with a variety of biographical details)”*(p. 227). In particular, he notes that it acts as a symbolic reference point for other mental contents as well as providing a self-centered view of the world so that objects and events are seen from the perspective of the organism that the self symbolizes. We suggest that this embodied “self” forms the basis for the idea that we own both our mental processes and our embodied form and “*with the assistance of past memories of objects and events, we can piece together an autobiography and reconstruct our identity and personhood incessantly*“ (Damasio, 2003, p. 277).

The creation of a stable executive reference system, the “self” (Prinz, 2003), is central to our non-executive account of “consciousness” where we see it as another strategic high level product of non-conscious CES systems offering as it does a critical focus point for the personal narrative. In other words, the embodied self or “center of narrative gravity” (Dennett, 1991) is a conduit for internal broadcasting and an attributional locus for executive capacity including control over psychological functions. As such, it provides a consistent, coherent gravitational center, and reference point for all externally broadcasted contents of the personal narrative and subsequent wider social interaction. The embodiment of “self” as an independent agent in the world is a developmentally evolving mental representation, which we suggest stems from a form of inherited archetype, similar to the self-acquisition device posited for language development (Chomsky, 1965). Seen as the product of non-conscious CES systems, the construction of self pervades the internally broadcast narrative with a focus, unity, continuity, and consistency over time, while also serving to integrate perception and memories (Sui and Humphreys, 2015). Consequently, any disturbance to the development, or normal operations of the internally represented self can result in anomalous subjective experiences such as the depersonalization and disturbed self-other/self-world boundaries seen in schizophrenic spectrum disorders (Mishara et al., 2016). It is arguable that our brains can generate alternative self-related narratives reflecting among other things different social roles we enact in our lives and that these may compete for entry into the personal narrative by the CES depending on the ongoing task.

We agree with Dennett (1991) that the creation of self as a representation comprises part of a survival tactic, analogous to a spider spinning a web, in which we develop a story to inform others, as well as ourselves, of who we are—and “*just as spiders don't have to think, consciously and deliberately, about how to spin their webs ……. (we) do not consciously and deliberately figure out what narratives to tell and how to tell them. Our tales are spun, but for the most part we don't spin them; they spin us*.” (p. 418)

It is important to note that our account does not challenge the significance and theoretical importance of current concepts of “self-awareness” (“self-consciousness” in traditional terminology) and “self-image” but rather places the processes and constructs they refer to as products of non-conscious systems mediated by the CES and reflected in the personal narrative. Our model proposes that they do not depend on, or require, a collateral “experience of consciousness” (personal awareness).

## Solving the “hard problem”?

The hard problem (Chalmers, 1996) involves two questions: First: “How and why do neurophysiological activities produce the “experience of consciousness”?”. Our account addresses this by concluding that personal awareness is a passive, emergent property of the non-conscious processes that generate the contents of the personal narrative and is not causally or functionally responsible for those psychological contents. The converse question “How can the non-physical experiences of “conscious awareness” control physical processes in the brain?” is consequenctly no longer relevant. We propose that there are no top-down executive controls exerted by either personal awareness or the personal narrative as both are psychological end-points of non-conscious processes.

## A “new hard problem”

A major challenge for the future lies however in the discovery of the neural mechanisms underlying personal awareness, though in our view this will not reveal its purpose—just as understanding the physical mechanisms involved in the creation of rainbows or eclipses does not provide an explanation as to their purpose. Nevertheless, as with rainbows and eclipses, it will be satisfying to eventually understand the neural processes behind it. In particular, we need to explore the association of personal awareness with particular types of information processing and whether this is unique to neural systems or can also be created in inanimate systems. However, this future challenge lies within the interdisciplinary domains of physics, philosophy, neuroscience, and information processing rather than cognitive science alone.

A related problem for any line of research that takes personal awareness as its focus is that of devising an objective means of determining its presence. Currently, we infer the existence of personal awareness in others by virtue of a commonality we share in belonging to the same species and having the same neural apparatus and mental states. We can determine the ongoing content of an individual's personal narrative by requesting a verbal report but this does not confirm the presence of personal awareness. If we ask “are you aware of this” and the answer is affirmative, we are inclined to readily accept this as confirmation of the “experience of consciousness”—the age-old philosophical question is whether we would draw the same conclusion if this response was elicited from an inanimate information processing system or indeed was signed by a non-human primate.

## Evolutionary benefit

So is there a purpose of “consciousness”? In our view, given the analogy with the rainbow, pursuing this question is liable to lead to confusion and there is no evolutionary benefit associated with personal awareness *per se*—it is simply the phenomenological accompaniment to the non-consciously mediated personal narrative. The personal narrative, however, we would argue has significant adaptive purpose for the individual and even more significant social evolutionary advantage, given the ability of individuals to transmit selected contents of their personal narrative to others via the process we have labeled External Broadcasting (see Figure 1). Our account is also broadly consistent with the views of others (Nietzsche, 1974; Jaynes, 1976; Humphrey, 1983; Barlow, 1987; Dunbar, 1998; Charlton, 2000; Velmans, 2000; Prinz, 2006; Frith, 2007, 2010; Baumeister and Masicampo, 2010) who accept that any evolutionary advantage lies not in the “experience of consciousness” (personal awareness) itself, but in the ability of individuals to convey selected aspects of their private thoughts, beliefs, experiences etc. to others of their species. We see personal narratives as having evolved over time and we assume they may not have always been accompanied by personal awareness in their early stage of development. However, at a certain level of computational complexity we assume the parallel quality of the subjective experience (the rainbow) became more evident, and in need of an explanation. An obvious response to the latter, given the temporal contiguities involved and the development of a gravitational self, was the attribution of causal or agentive properties.

Specifically, we regard External Broadcasting as a natural competence of all humans (and some animals) selectively to convey (Internally Broadcast) private psychological contents of the personal narrative (thoughts, ideas, concepts and abstractions, including art and music), as well as experiences (sensations, feelings, urges, concerns etc.), to others, predominantly via gestures and speech. The construction of a personalized identity (the self) by non-conscious systems representative of the “author” of this externally broadcast narrative content, including the attribution of the psychological qualities of awareness and agency, provides for a coherent reference point. The selection of contents of the internally broadcast narrative for External Broadcasting is controlled by the CES within the broad remit of communicating a personalized task-relevant account of current ongoing perceptions, thoughts, ideas, plans etc. to others whilst ensuring that the individual's self appears purposeful and consistent over time within the context of expectancies and beliefs of the immediate social group.

This process is far from linear, with a second or possibly multi-stage process within the CES working to monitor,correct and amend earlier transmitted content during and after external broadcasting. Hence, non-consciously generated slips of the tongue that we all experience and the often subsequent, equally non-consciously generated, “I'm sorry that came out wrong—what I meant to say was…….”. More importantly, the non-conscious processes within the CES generating the personal narrative have access to the externally broadcast outputs of others, as well as the individual's own previous written or digitized outputs. This is important for the future behavior and cognitions of the individual, but also by re-transmission (re-tweeting), via their non-conscious systems, into the personal narratives of others has the potential in turn to influence their future thoughts and ultimately their behavior.

A second supra-individual level of transmission, *Cultural Broadcasting* (see Figure 1), is achieved via artifacts, writing, books, art, music, and more recently through radio, television, social media, and films, creating a pool of knowledge, skills, ideas, and beliefs potentially accessible to all members of the species. Ultimately, shared information and beliefs are shaped through Cultural Broadcasting into autonomous self-sustaining social systems traditionally embodied in education, art, social norms, and laws, and in long-term physical systems such as libraries and museums. Internal and External broadcasting as well as access to cultural resources may confer some survival advantage for the individual, but the major evolutionary driver and beneficiary is the group-benefit conferred by the process of Cultural Broadcasting and the establishment of an autonomous, supra-individual pool of culturally based-resources.

In this section and others that follow, where we use the established terms “mind,” “contents of mind,” “mind-reading” etc—it is important to underline that within our model, all of these refer to non-conscious processes and constructs. In social contexts, non-conscious systems orchestrate the external transmission of selective contents of the personal narrative, allowing the knowledge and perspective of individuals to be shared more widely with others in the group. This facilitates the fluidity of co-operation, sharing of information, and the development of adaptive strategies, as well as the construction of a Theory of Mind and the attribution of an awareness of self to others, at both an individual and cultural level (Humphrey, 1983; Aktipis, 2000; Charlton, 2000; Frith, 2007, 2010; Graziano and Webb, 2017).

The individual, social, and cultural significance of the development of a Theory of Mind, particularly through pretend play as a basis for “mind reading” is increasingly recognized and the failure to do so at an individual level can be related to autism (Baron-Cohen, 1995; Frith and Happé, 1999; Heyes and Frith, 2014). In addition to the potential for predicting and influencing the thoughts and behaviors of others, there is a broader social dimension via cultural broadcasting of beliefs, prejudices, feelings, and decisions originating in non-consciously generated personal narratives. This in turn, raises the possibility that the mental content of individuals can be changed by outside influences such as formal education, new forms of social media, and music. The broadcasted or communicated narrative allows humans to take a shared-view, rather than an exclusively self-referential view. Revealing the content of our personal narrative to others: including our beliefs, prejudices, feelings, and decisions allows group members to characterize others and generate strategies, such as predicting their behavior, in particular through the capacity for “mind reading” (Heyes and Frith, 2014), all of which is potentially beneficial for social or species survival.

Communicating the contents of the personal narrative is also importantly a means of disseminating ideas that can be incorporated into social systems including the widespread, well-recognized concepts of free-will and natural law. Indeed, given their cultural prominence in most social and democratic cultural systems, it seems likely that these are significantly embodied in non-conscious systems for social adaptive advantage. Importantly, the social sharing of personal narratives allows for the possibility that their content can also be changed, again via non-conscious systems, by outside influences such as education and socializing.

At a cultural level, norms and values generated through individual interaction compete in society as “memes” that service the process of cultural evolution (Dawkins, 1976; Plotkin, 1994; Blackmore, 1999). It is inevitable perhaps that competition between memes has on occasions led to conflict and bloodshed, but on balance the outcomes in the form of social constructs such as democracy, human rights, equality, socialism, and capitalism, can be regarded as beneficial and species-enhancing. None of the social systems that human societies depend on are possible, however, without the smooth and consistent ability to share the contents of individual personal narratives.

## External broadcasting: the social role; hypnosis and suggestion

Contained within external broadcasts can be direct verbal suggestions (including hypnotic suggestions) that can influence a range of psychological phenomena, including so-called “automatic” processes, in the recipients and which may relate to a socially adaptive human trait (Halligan and Oakley, 2014; Terhune et al., in press). As an example, it is widely accepted that perception involves a constructive process than relies on non-conscious inferences based on past experience and prior knowledge (Gregory, 1997) and that as a consequence, we as individuals cannot, for example, change our perception of the colors in a Mondrian picture by the exercise of voluntary intention or choice. However, this colorful display can be turned into a gray scale image by appropriate suggestions, particularly in highly hypnotically suggestible individuals (Kosslyn et al., 2000; McGeown et al., 2012). In a recent study, Lindeløv et al. (2017) have shown, in a randomized actively-controlled trial, that working memory performance can be effectively restored by suggesting to hypnotized brain injured patients that they have regained their pre-injury level of working memory functioning. Phenomena of this sort have led to the increasing use of hypnosis with direct verbal suggestion as a tool in cognitive research as well as being a topic of interest in its on right (Oakley and Halligan, 2009, 2013; Oakley, 2012; Halligan and Oakley, 2013; Landry and Raz, 2016; Terhune et al., in press). Hypnosis-based research, including Kihlstrom's classic “The Cognitive Unconscious” paper (Kihlstrom, 1987), has been influential in developing our model.

The wider significance of these studies is that, whilst the effects of hypnotic suggestion can at first sight appear extraordinary (i.e., beyond that which would be expected), direct verbal suggestibility is normally distributed in human populations and can be seen as a prime example of a broader socially adaptive trait that is powerfully capable of harnessing aspects of our non-conscious systems (Halligan and Oakley, 2014). Consistent with this, *empathy* is one of the few personality traits correlated with hypnotic suggestibility (Wickramasekera and Szlyk, 2003) and is also associated with the ability to share at second hand an experience such as pain with another (Singer et al., 2004). On this basis, one plausible explanation for the widespread ability to respond to verbal suggestion is that suggestion underlies a socially cohesive ability to indirectly share experiences by re-creating them in others, not dissimilar to the function of “mirror” neurons that fire both when an animal acts and when the animal observes the same action performed by another.

In a similar vein it has been proposed that the often neglected psychological capacity of suggestibility has a more powerful social impact as a means of transcending reality (Schumaker, 1991) and understanding the minds of others, as well as promoting attachment and other cohesive social processes (Ray, 2007; Halligan and Oakley, 2014). It has also been noted that experiences similar to those produced in response to hypnotic suggestion are seen cross-culturally associated with religious and spiritual beliefs and practices, again indicating an important sociological function (Dienes and Perner, 2007). Related to this, hypnotic suggestion has been shown in fMRI studies to reliably produce experiences of alien control, thought insertion, and automatic writing seen in spirit possession, mediumship, and shamanism (Deeley et al., 2014; Walsh et al., 2014).

## Suggestion, dissociation, and related clinical conditions

One advantage of our account is that it provides a potential framework for explaining several enigmatic phenomena such as, suggestibility, dissociations between implicit and explicit awareness and dissociative phenomena more generally. As noted above, all humans are responsive to some extent to direct verbal suggestion, typically contained within the external broadcast from another individual, and this responsiveness may reflect a socially adaptive trait. The most widely researched example is hypnotic suggestion, where the suggestion (defined as a communicable belief or perception) is delivered following a hypnotic induction procedure (Halligan and Oakley, 2014). According to our account, congruent responses to an external direct verbal suggestion result from non-conscious systems in the recipient's brain being recruited to engage in a socially-driven role-play by creating neural activity consistent with the suggested change itself (Oakley and Halligan, 2009, 2013). As a result, suggested experiences become part of the recipient individual's internally broadcast personal narrative, and concurrently also part of their personal awareness, and so are experienced as real, albeit involuntary, events. For example, suggested, but not imagined, experiences of pain are accompanied by activity in brain areas involved in pain processing (Derbyshire et al., 2004).

Similarly, involuntary hand movements following the suggestion that the hand is being moved passively by a pulley show the same patterns of neural activity as an actual passive movement (Blakemore et al., 2003) and when limb paralysis is suggested, but not when it is feigned, there are inhibitory changes in related motor areas similar to those seen in a hysterical limb paralysis (Halligan et al., 2000; Ward et al., 2003; Deeley et al., 2013a). Within the personal narrative, the account is of an actual primary experience, with the suggested effects being recorded, and reported, as involuntary. Interestingly, a record of hearing the suggestion itself may also be part of the personal narrative, unless the original suggestion includes source amnesia. It is important to emphasize that, in our model, direct verbal suggestion is seen as being received (via external broadcasting) and processed via the recipient's non-conscious sensory systems. As a result, brain states congruent with the suggestion are generated by central executive structures in accordance with the externally directed role-play. The results of this process are then broadcast into the personal narrative by central executive structures with the accompanying, parallel conscious experience. Consequently, the process initiated by a direct verbal suggestion is entirely bottom-up in its execution.

The idea of a non-conscious, motivated role-play underlying the effects of external suggestion also provides an explanation for some clinical conditions with the caveat that the “suggestion” or false belief (delusion) may be generated internally by non-conscious systems (Halligan, 2011). Consistent with this, hypnotic suggestion has been used to create experimental analogs for internal voices (hallucinations) and passive (alien) or unwilled (anarchic) movements seen in clinical conditions such as schizophrenia and in the culturally driven experiences of thought insertion and automatic writing (Blakemore et al., 2003; Deeley et al., 2013a,b, 2014; Walsh et al., 2014, 2015b), as well as to create delusions and disorders of belief (Cox and Barnier, 2010; Connors, 2015), such as those underlying the inability to recognize one's own reflection (Connors et al., 2013) and the transformation of gender identity (Noble and McConkey, 1995).

In motor conversion disorder (hysteria), as in hypnosis, the observed dissociative symptoms of paralysis, aphonia etc. are not related to known physical or physiological damage but rather are represented as subjectively powerful, “real” phenomena within the personal narrative (Oakley, 1999a; Bell et al., 2011). Even more dramatic perhaps, as a partial analog of dissociative identity disorder (multiple personality), is the phenomenon of the “hidden observer” in hypnosis (Hilgard, 1977) in which a parallel narrative process is suggested, classically in an individual concurrently experiencing suggested analgesia (Hilgard et al., 1975). This dissociated second narrative state can then be cued to represent the feeling of pain in the personal narrative, returning to the analgesic state narrative when the cue is reversed. The “hidden observer” reflects the existence of a second narrative process relating to a single self-representation. In dissociative identity disorder, two (potentially more) representations of self with their associated histories and ongoing experienceses are available for entry into the personal narrative. Importantly, again all of the above implicate bottom-up, rather than top-down, influences on the streams of non-conscious processes that contribute to the content of the personal narrative and consequently to the parallel conscious experience. Specifically, where direct verbal suggestion is involved, the influence arises via a spoken input, is processed low down in the hierarchy of brain processes receiving and analyzing speech, resulting in changes within non-conscious systems that may ultimately be reflected in the recipient's personal narrative (with the accompanying personal awareness).

## Free-will and personal responsibility

The commonly assumed belief in “free-will” (i.e., a self-directed “voluntary” ability to make non-deterministic, non-random choices between different possible courses of action) has long been considered a hallmark and function of “consciousness” and of “conscious awareness” in particular (e.g., Pierson and Trout, 2017). However, there seems no reason to suppose that this ability is beyond the processing capacities of fast-acting, non-conscious brain systems. If, as we propose, personal awareness, with its ubiquitous sense of self, agency, and decision making, is a accompaniment to underlying psychological processes, what implications does this have for socially-revered concepts of free-will and personal responsibility?

In support of the construct of free-will, it is sometimes argued that, although there is evidence that awareness of the intention to make a movement occurs later than the preparatory neural activity, the act of countermanding the previously experienced intention demonstrates the active involvement of a higher-level “conscious” process (i.e., an exercise of “conscious” free-will). According to our account, any subsequent decision or action to countermand a previously intended movement (for whatever attributable reason), can just as easily be explained as being generated by the same non-conscious systems (equally as an act of free-will) but with the “countermanding intention” only being broadcast temporally later into the personal narrative.

As our account removes any self-serving controlling influence from the contents of the personal narrative and personal awareness, it could be seen to undermine the principle of personal accountability. We, however, consider personal responsibility, a mainstay of the cultural broadcasting architecture and a social contruct critical to most democratic and legal systems, as lying within non-consciously-generated actions and intentions transmitted into the personal narrative and in particular where these same contents have been publicly announced via external broadcasting. Both of these events are accompanied, albeit passively, by personal awareness (“experience of consciousness”)—thereby meeting the traditional moral and legal benchmark.

In our account, everyday constructs such as free-will, choice, and personal accountability are therefore not dispensed with—they remain embedded in non-conscious brain systems where their existence as near universal constructs serving powerful social purposes could well be seen in large part to be a consequence of cultural broadcasting impacting on personal narratives.

## Conclusions

Historically compelling folk and lay accounts assume that “consciousness” provides for some executive control over the psychological processes that populate much of our mental content. This largely unquestioned and intuitively appealing view has received numerous challenges over the past 30 years. Even the most scaled-back accounts, however, appear reluctant to abandon completely the attribution of some kind of executive role to “consciousness.” Overall, these traditional accounts distinguish two main components: the “experience of consciousness” and the “contents of consciousness,” which we refer to as “personal awareness” and a self-referential “personal narrative.”

We take no issue with the experiential primacy or reality of personal awareness and the related powerful sense of agency and self, that we all feel. We argue, however, that central to the traditional domain of “consciousness” is a personal narrative created by and within inaccessible, non-conscious brain systems where personal awareness is no more than a passive accompaniment to this process. In this view, both the personal narrative and the associated personal awareness are end-products of widely distributed, efficient, non-conscious processing that arrives too late in the psychological process cycle for there to be a reason to infer the necessity of an additional independent executive or causal capacity to either of them.

As far as our model is concerned, the contents of the personal narrative are end products of non-conscious systems. The fact that personal awareness (Huxley's steam whistle) accompanies the contents of the personal narrative is causally compelling but not relevant to understanding and explaining the psychological processes underpinning them (Huxley, 1874). We have argued that the everyday perception/belief of a casual association between the “experience of consciousness” and the “contents of consciousness” is based on a longstanding, albeit understandable, misattribution/misconception. We nevertheless accept that the non-conscious processes involved in creating the personal narrative may also create the experience of consciousness, much in the way that the hidden processes of reflection, refraction, and dispersion of light from water droplets generate the perception of the rainbow. In terms of our account, the “hard question” of how consciousness can influence brain processes is not so much “hard” as simply “wrong.” We are left with the reverse, equally “hard” but, from a cognitive psychological perspective, not theoretically relevant question of how non-conscious processes creating the personal narrative also appear to create an experience of consciousness.

While our account does not deny the reality of personal awareness or its association with personal narrative contents, we conclude that considering personal awareness as a form of high level executive psychological process has hindered the understanding of the nature and structure of the more relevant underlying psychological systems. The proper focus for both research and theory going forward is those neuro-psychological processes that underly the personal narrative, which represents a continuously updated, self-related, meaningful, and selective account of on-going activity created by and within non-conscious systems. The personal narrative account informs historically consistent behavior in ongoing situations, provides potential content for retention in autobiographical memory and defines the self-related information available for communication to others. This is congruent with the view that autobiographical/episodic memory is not a record *of* events *per se* but is a partial and selective record of a personalized narrative *about* events.

As a real, but essentially non–executive, emergent property associated with the selective internal broadcasting of non-conscious outputs that form the personal narrative, we consider personal awareness to lack adaptive significance in much the same way as rainbows or eclipses. The non-consciously generated personal narrative on the other hand forms the basis for both significant individual and social adaptive advantage. The main evolutionary advantage, lies in the selective public transmission of contents of the personal narrative, again under the control of non-conscious systems, and the sharing of these essentially private contents (thoughts, feelings, and information) with others in the local and wider social group. As part of this adaptive process, individuals are predisposed not only to transmit information from their own personal narrative but also to receive and process the externally and culturally transmitted outputs from others. In becoming available to others the broadcast (and re-broadcast) content of individual personal narratives supports the mutual understanding of the drivers behind thought and behavior. This in turn facilitates the dissemination of ideas and beliefs, and ultimately the construction of resilient supra-individual social, cultural, and legal systems which has contributed to the stability and evolutionary adaptedness of the species.

## Author contributions

Both authors listed have contributed equally to the work and approved it for publication.

### Conflict of interest statement

The authors declare that the research was conducted in the absence of any commercial or financial relationships that could be construed as a potential conflict of interest.
